# Coreferentiality: A New Method for the Hypothesis-Based Analysis of Phenotypes Characterized by Multivariate Data

**DOI:** 10.1371/journal.pone.0033990

**Published:** 2012-03-29

**Authors:** Constantin Fesel

**Affiliations:** Instituto Gulbenkian de Ciência, Oeiras, Portugal; University of Leuven, Rega Institute, Belgium

## Abstract

Many multifactorial biologic effects, particularly in the context of complex human diseases, are still poorly understood. At the same time, the systematic acquisition of multivariate data has become increasingly easy. The use of such data to analyze and model complex phenotypes, however, remains a challenge. Here, a new analytic approach is described, termed coreferentiality, together with an appropriate statistical test. Coreferentiality is the indirect relation of two variables of functional interest in respect to whether they parallel each other in their respective relatedness to multivariate reference data, which can be informative for a complex effect or phenotype. It is shown that the power of coreferentiality testing is comparable to multiple regression analysis, sufficient even when reference data are informative only to a relatively small extent of 2.5%, and clearly exceeding the power of simple bivariate correlation testing. Thus, coreferentiality testing uses the increased power of multivariate analysis, however, in order to address a more straightforward interpretable bivariate relatedness. Systematic application of this approach could substantially improve the analysis and modeling of complex phenotypes, particularly in the context of human study where addressing functional hypotheses by direct experimentation is often difficult.

## Introduction

Biological and biomedical research has undergone an unprecedented evolution of technologies in recent years, to a substantial part due to techniques that yield highly multivariate phenotype data such as microarray-based RNA expression analysis. Techniques to acquire proteomic, serologic, cytometric and other data show similar tendencies toward high-throughput methods and therefore high-level multiparametricity. Currently used methods of data analysis, however, are far from using the full information depth of such data. This may be best exemplified by genome-wide genetic association studies (GWAS), which are generally unable to use the largest part of their theoretically available information due to excessive multiple testing that leads to high false-positive (type 1) error rates. Correction of resulting *p*-values for this multiple testing unavoidably obscures results that do not reach extremely high significance levels. The same problem principally appears in the analysis of multiparametric phenotypic data, where individual variables are in most cases also tested one by one, e.g., in mRNA expression analysis with the aim to find the most over- or underexpressed genes.

It is classic textbook knowledge that genuinely multivariate data analysis has the principal capacity to avoid the error rate inflation and loss of power caused by such multiple testing (see e.g. [1], chapter 5.1). It does that by testing a hypothesis with only one statistical test that is designed on the basis of a model that integrates multiple variables taken from the same units of observation. Classic multivariate approaches such as multivariate regression, canonical correlation or principal component analysis, however, do not offer practically straightforward solutions for many problems. Therefore, specific tayloring of multivariate statistical tests for particular types of hypotheses can be useful to make multivariate approaches easier applicable as well as to improve the interpretability of results. This paper proposes such a novel statistical test for a specific hypothesis type.

The principal strategy of multivariate statistical testing is to bring multiple variables into a predefined context that represents a hypothesis of interest. A classic way to do this is to relate two separate sets of multivariate data to each other in a multivariate regression model. This allows to study whether and to what extent one set of variables can be explained by the other. However, this question does not always represent hypotheses of interest, which are not necessarily well represented by a multivariate dependency model. Particularly when incompletely characterized complex systems are explored where measurable variables are mainly defined by practical accessibility and direct causal effects are largely unknown, it is usually inadequate and overambitious to model explicit dependency structures. This is the case in many biological and biomedical research contexts. In such contexts, useful hypotheses can sometimes still be naturally formulated as relations between already characterized specific effects on a complex phenotype as a whole that is characterized by multiple other variables. The statistical problem to solve here is not the explanation of these other variables and their dependency structure, but rather to assess the relation between the specific effect variables. A criterion to test this could be whether two test variables parallel or resemble each other in their relatedness to an independently obtained multivariate data set that represents a context phenotype. Since it is neither necessary nor intended to model causal effects, a most adequate test statistic should be independent of any dependency modeling and therefore resemble the strategy of classic bivariate correlation rather than regression analysis. The author is not aware of any existing statistical criterion or test of this type. Here, an appropriate criterion is proposed together with a way to test it statistically: coreferentiality.

Avoiding any direct testing of variables obtained as part of a large multiparametric data set, the coreferentiality approach rather uses them as reference data to ask the question: will two test variables separately obtained for the same sample parallel each other in their respective relations to the reference data ? This indirect relatedness of the two test variables indeed represents a hypothesis of the same type as it underlies the testing of their simple bivariate correlation. However, instead of the correlation hypothesis ‘the higher (or alternatively, lower) one variable is, the higher is also the other variable’, the coreferentiality hypothesis states that ‘the more one variable correlates (in one or the other sense) with a respective reference variable *Y*_i_, part of the reference data set **Y**, the more also the other variable does that’. This is equivalent to both variables parallelly referring, i.e., co-referring to the reference data. Coreferentiality is expected if and only if at least a subset of the reference data is related to a hypothesized effect that also relates the test variables to each other. The directedness of this effect is in principle irrelevant since correlation is invariant to whether and how underlying causalities are directed. If the reference data are phenotypic, coreferentiality will reflect the functional relatedness of the test variables in respect to this multivariate phenotype, naturally including possibly complex bidirectional dependencies. Coreferentiality is absent (a) when the test variables are not related, as well as (b) when an effect that relates them exists but does not affect the reference data. The latter implies that correlated variables are not necessarily coreferential. Neither are coreferential variables necessarily correlated: it is possible that they parallelly relate to the reference data without showing a direct correlation. This is in fact expected in a condition of particular interest that is not straightforward to test conventionally: when the two test variables represent effects with unrelated origin but related functionality toward a phenotype.

Taken together, coreferentiality appears as an interesting test criterion, particularly to explore complex phenotypes characterized by multivariate data under functionally defined hypotheses that can be formulated as a relation of two variables. The practical requirement that has motivated this work occurred when the author and others studied relations of cytokines, regulatory T-cells and specific disease-associated antibodies in respect to multivariate autoantibody profiles in patients with Systemic Lupus Erythematosus and their unaffected relatives [2]. This study, to be published in conjuction with this paper, may further exemplify how coreferentiality can be practically used in a concrete research context.

In the following, corefentiality will be mathematically defined, a statistical test for it described and its power of detection and specificity assessed for diverse conditions in terms of sample size, number and informativity of the reference variables as well as the direct correlation between the test variables.

## Results

Coreferentiality is defined as the parallellity of correlations of two variables *X_1_* and *X_2_* with a set of reference variables **Y** = {*Y_1_*, *Y_2_*, *…*, *Y_k_*} drawn from the same sample, and *X_1_* and *X_2_* are coreferential to the degree that 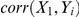 correlates with 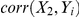. Accordingly, *X_1_* and *X_2_* can be called truly coreferential if the ***coefficient of coreferentiality*** between *X_1_* and *X_2_* in respect to **Y**, 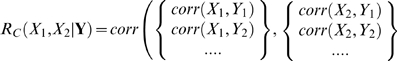, differs from its expected value *R_C_*_0_ occurring if 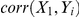 and 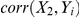 are uncorrelated. Thus, coreferentiality can be shown by rejecting the null hypothesis **H_0_** that 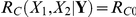. *R_C_*_0_ is not necessarily equal to zero since (a) correlations between *X_1_* and *X_2_* and (b) structures within the **Y** data can influence it. Particularly for correlated *X_1_* and *X_2_*, *R_C_*_0_ markedly differs from zero (see below). **H_0_** can be tested by the probability that an observed *R_C_* or more extreme value occurs in a (null) distribution of *R_C_*_0_, i.e., *R_C_* values expected in the absence of non-random correlations between *X* and *Y* variables while 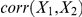 and 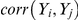 are preserved. Such a null distribution can be generated by random permutations of true data, following the adaptation of the classic randomization theory [3], [4] for linear correlations [5]. In particular, a null distribution with the properties to test **H_0_** can be generated from *R_C_* values calculated from random permutations of the true *X_1_*, *X_2_* and **Y** data where *X_1_* and *X_2_* are parallelly reshuffled against the **Y** data left in place, a procedure that is invariant against both 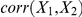 and 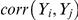. An empiric *p*-value can then be determined by the proportion of permutations giving the observed or a more extreme absolute value of *R_C_*, corresponding to a two-tailed test that was shown to closely follow the results of standard testing of Pearson's *R* [5].

Accordingly, in order to test the significance of coreferentiality, 1000 permutations were here generated from a respective data set by parallelly reshuffling *X_1_* and *X_2_* against the **Y** data, and a corresponding empiric *p* was calculated by the proportion of permutations that yielded an *R_C_* value with its absolute exceeding the absolute *R_C_* of the true data. Using this test, power and robustness of coreferentiality testing were assessed in simulated coreferential data with defined properties. First, *X_1_* and *X_2_* were simulated as two uncorrelated (*R*<0.01) sets of Gaussian distributed random numbers N(0,10) with sizes N varying between 50 and 500. Then, reference data *Y_1_*, *Y_2_*, …, *Y_k_* consisting of *k* = 130 variables were generated with sizes equal to *X_1_* and *X_2_* and values assigned to them by linear combinations of *X_1_*, *X_2_* and Gaussian-distributed noise: 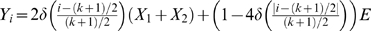, with *E* being random numbers (Gaussian noise) distributed N(0,10) as *X_1_* and *X_2_*. In this formula, **Y** data were designed so that *X_1_* and *X_2_* contributed to them with equal weights, these weights being defined by their average absolute degree of determination *δ*, corresponding to relative degrees of determination varying among the *Y_i_* along a linear gradient from −2*δ* to +2*δ*.

**Figure 1:**
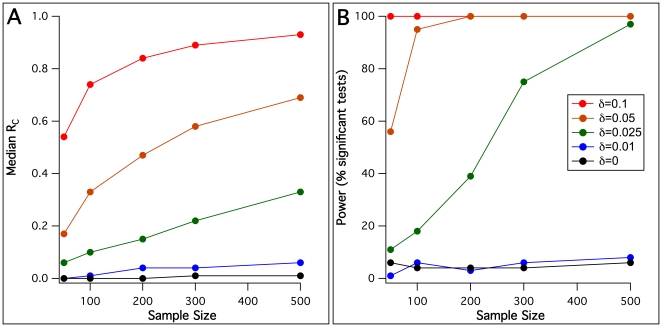
Coreferentiality coefficients and power of coreferentiality detection with uncorrelated test variables *X_1_* and *X_2_*. For each possible combination of various sample sizes and reference data dependency degrees *δ* (see insert in panel B), 100 data sets were simulated and tested with the permutation test described. Median coreferentiality coefficients *R_C_*. B. Power of detection using the permutation test described (percentage of test *p*<0.05).

First, the power to detect coreferentiality was addressed. Particularly, 100 simulations were generated for each of the 25 combinations of five different sample sizes and five different values of *δ*. Sample sizes included N = 50, N = 100, N = 200, N = 300 and N = 500; *δ* values were 0, 0.01, 0.025, 0.05 and 0.1, corresponding to average degrees of determination from 1–10% and *δ* = 0 as a negative control simulation where the reference data were unrelated to *X_1_* and *X_2_*. For each individual data simulation, the coefficient of coreferentiality was determined and tested by the described permutation-based significance test. Fig. 1 shows the resulting median coreferentiality as well as the power of detection (in terms of the percentage of significant tests at the level *p*<0.05) for each condition. It can be seen that coreferentiality coefficients steadily rose with both sample size and *δ*. An average determination of the reference data of 2.5% by each test variable was sufficient to detect the simulated coreferentiality in 500 samples with >95% power. For 5% and 10% average determinations, the same power was already reached with 100 and 50 samples, respectively. However, 1% average determination was not sufficient to detect coreferentiality in the simulated conditions, with power values indistinguishable from *δ* = 0.

The *δ* = 0 condition, i.e., negative control simulations with no coreferentiality, furthermore served to assess the specificity of the test. The conventionally used 5% significance level, applied here as well, predicts that an expected rate of false-positive tests of 5%. The average percentage of tests reaching this significance level in the applied test in the five conditions tested with *δ* = 0 was 4.8%, thus demonstrating satisfactory specificity and no detectable *p*-value inflation.

**Figure 2:**
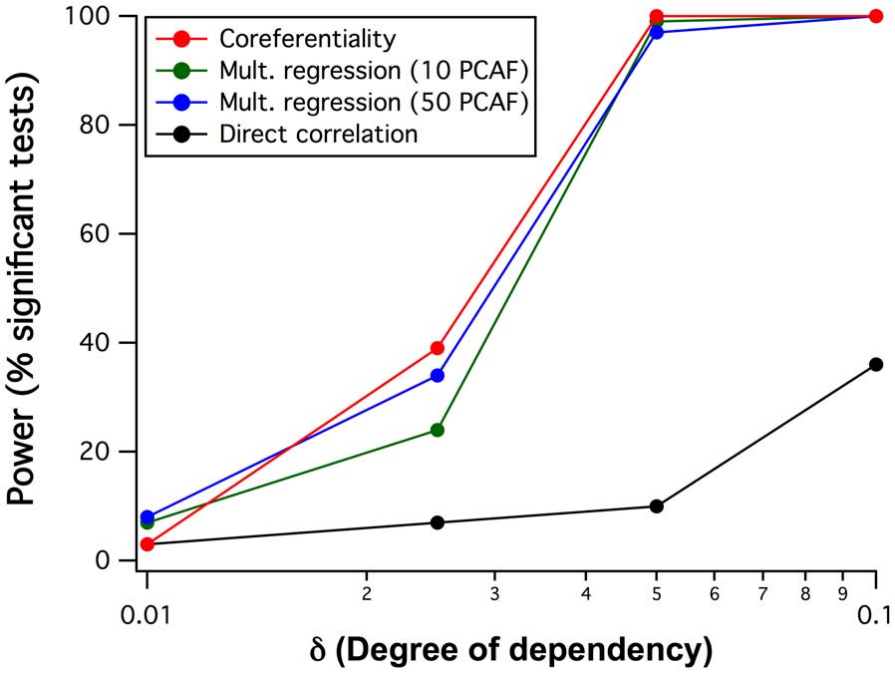
Comparison of the statistical power to detect coreferentiality, dependency in multiple regression, and classic correlation. The power of each method was tested in 100 respectively simulated data sets, all with a sample size of 200 and different reference data dependency degrees *δ*. Multiple regression was tested for a given test variable *X* in dependency on the scores of either 10 or 50 PCA factors derived from the 130 reference variables. Bivariate correlation was tested for simulations with *X_2_* depending on *X_1_* with the degree *δ*, and tested by simple linear regression.

It appeared of interest to compare the detected power of the coreferentiality test with related statistical methods even if they address different questions. Therefore, 100 data simulations were generated under the same conditions as previously, to assess the power of classic multiple regression relating the test variables *X* to **Y**. Since multiple regression analysis with all 130 reference variables was not always feasible due to collinearity, principal components were derived from all *Y*_i_ in each simulation and linear multiple regression analysis performed between each *X* and either 10 or 50 principal components. The power of both calculations in terms of the frequency of tests significant at the 5% level, for the five *δ* levels mentioned and N = 200, is depicted in Fig. 2 and compared with the power of coreferentiality testing. It turned out that both methods had comparable power, and that coreferentiality was even slightly more powerful. Finally, to compare these results with a classic two-variable test, 100 further simulations were generated where *X_2_* was directly partially dependent on *X_1_* with a degree of determination defined by *δ*: *X_2_* = *δX_1_*+(1−*δ*)*E*, and simple bivariate linear regression analysis performed for each simulation. As expected, this test was clearly less powerful (Fig. 2, black line), requiring an about 4-fold higher *δ* to reach the power of the multivariate tests.

**Figure 3:**
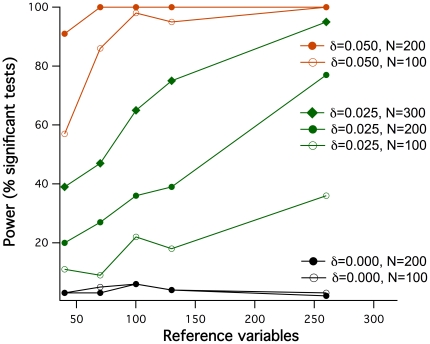
Power of coreferentiality detection in respect to the number of reference variables used. 100 respective data sets were simulated for different sample sizes N, different reference data dependency degrees *δ* (see insert) and either 40, 70, 100, 130 or 260 reference variables, and tested for coreferentiality with the permutation test described. The occasional deviation from monotonous behavior in the curve representing *δ* = 0.025, N = 100 is due to stochasticity.

Apart from *δ* and sample size, also the number of reference variables *k* was expected to influence the power of coreferentiality testing. Therefore, further sets of data simulations (100 per condition as throughout this description) were generated with *k* ranging from 40 to 260, combined with different *δ* values and either N = 100 or N = 200, and tested for coreferentiality. The results, depicted in Fig. 3, show that the power indeed markedly increased with *k*, over long ranges aproximately linearly. They furthermore indicate that particularly in conditions with limited power such as *δ* = 0.025, substantially higher power can be reached with identical *δ* and N, solely by increasing *k*. However, this is only the case when the reference variables remain equally informative. In contrast, adding noninformative or biased reference variables can substantially reduce the power: when 130 unrelated reference variables were added to 130 informative ones, the power of detecting coreferentiality was clearly lower than when only the 130 informative variables were used (54% versus 75% power with *δ* = 0.025, N = 300). An even more serious loss of power (24% power for *δ* = 0.025, N = 300) was observed when *X_1_* was itself included in the reference data as one of the *Y* variables, generating a correlation outlier in the reference data. Including both *X_1_* and *X_2_* as *Y* variables even abolished all power to detect coreferentiality in this condition.

**Figure 4:**
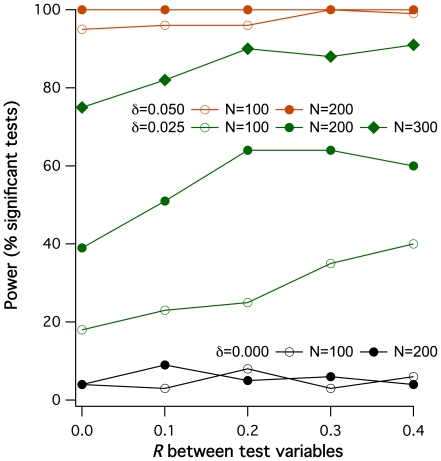
Power of coreferentiality detection in respect to the direct correlation *R* between the two test variables. 100 respective data sets were simulated for different sample sizes N, different reference data dependency degrees *δ* (see insert) and with defined correlations between *X_1_* and *X_2_*, ranging from 0 to 0.4. All simulations were tested for coreferentiality with the permutation test described, and for each included condition the power of detection at the 5% significance level was determined.

**Figure 5:**
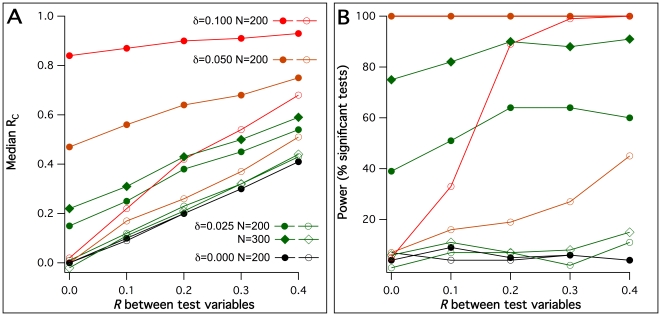
*R_C_* and power of coreferentiality detection with correlated test variables, and reference variables dependent on either one or both. For indicated dependency degrees *δ*, sample sizes N and correlations *R* between the test variables, 100 data sets were simulated respectively and tested with the permutation test described (lines with closed symbols according to the insert in panel A). The same was then repeated in the condition that reference data depended on only one test variable (lines with open symbols). A. Median coreferentiality coefficients *R_C_*. B. Power of detection using the permutation test described (percentage of test *p*<0.05).

All coreferentiality tests until here were performed with uncorrelated *X_1_* and *X_2_*. How does the coreferentiality test perform when they are correlated ? In simulations with *X_1_* and *X_2_* correlated by defined correlation coefficients *R* up to 0.4, shown in Fig. 4, *R_C_* values in fact defaulted not to 0 but to *R*. However, the permutation test was robust against these correlations as expected with *δ* = 0, i.e., when the reference data were unrelated to *X_1_* and *X_2_*, showing no more significant tests than the expected rate of false-positive ones (5.5%, 5.0%, 4.5% and 6.0% significant tests for *R* = 0.1, 0.2, 0.3 and 0.4, respectively). With *δ*>0, the detection power was slightly increasing with *R* until a plateau that depended on both *δ* and N. However, another question rose with *X_1_* and *X_2_* being correlated: coreferentiality can occur not only when both *X_1_* and *X_2_* are related to **Y**, but also when only *X_1_* is related to **Y** but *X_2_* sufficiently correlated with *X_1_* (or vice versa). To find out the probability of such ‘bystander’ coreferentiality, further sets of data simulations were generated and tested under the same conditions as above, but with only *X_1_* and not *X_2_* influencing **Y**. Results are shown in Fig. 5. It can be seen that ‘bystander’ coreferentiality is principally much lower in terms of *R_C_* than that observed when both *X* affect **Y** under equal *δ* and N. For *δ* = 0.025, there was furthermore no obvious deviation of the percentage of significant tests from the expected frequency of false-positive tests, so that the effect of ‘bystander’ coreferentiality in this low-effect condition appeared marginal. Only *δ* values of 0.05 and higher lead to substantial deviations from *R_C_* = *R* (Fig. 5A) and to relevant coreferentiality detection in terms of significant tests (Fig. 5B), actually up to 100% under *δ* = 0.1 and *R* = 0.4.

## Discussion

The coreferentiality approach described here, aiming to test whether two test variables relate to each other in respect to multivariate reference data, is basically new. The author is not aware of publications describing any method that would follow a similar approach. It could be shown that coreferentiality is robustly testable by an adapted permutation test, with a remarkable power comparable to multiple regression analysis and conserved specificity. Coreferentiality was not detected above the expected type-1 error rate in any condition where the test variables were unrelated either to each other or to the reference data. Addition of noninformative data or correlation outliers reduced the power, but did not inflate false-positive test results. Coreferentiality occurring due to direct correlation between test variables when only one was related to the reference data (‘bystander’ coreferentiality) was largely restricted to situations with strong dependency of the reference data, so that coreferentiality testing can indeed be said to detect primarily the indirect relatedness of two variables in respect to the reference data but not their direct relations. In this context it may be noted that it is possible to test and distinguish ‘bystander’ coreferentiality in empirical data, by assessing the probability of reaching/exceeding the test result of the true data with artificial secondary test variables simulated to be correlated only with the respective first test variable (applied in our accompanying paper [2]).

It remains to discuss the general applicability and usefulness of the coreferentiality approach. Currently, highly multivariate phenotype data as e.g. microarray-based RNA expression profiles are usually analyzed by two major approaches [6]. The first approach is to test the single readout variables separately, followed by an identification of the most informative ones. This approach is not only hypothesis-free but also bears the intrinsic problem of elevated type-1 error rates and according loss of power due to corrections for multiple testing, as it was discussed above. The second frequently used approach is to analyze the data by multivariate classification methods, usually clustering algorithms. This approach, also applied on top of the first one, is equally hypothesis-free and only allows to interpret results when the applied algorithm spontaneously leads to an interpretable classification under a simple criterion that has a high impact on the overall structure of the data. Since such classification criteria must be categorical, they are usually case-control or similar simple empiric discriminations, but do not represent functional hypotheses. Another multivariate approach that was previously followed by the author and others [7]–[17] is principal component analysis (PCA). This classic method does not use discrete classification as clustering does, but works strictly quantitatively and also allows an interpretation of how included parameters are combined in the “factor loads”. Representing a maximal proportion of the total data variance in a lower-dimensional subspace, however, PCA is genuinely hypothesis-free as well and designed to fit the phenotype data as they are, but not to explore them according to functional hypotheses.

Taken together, all these hypothesis-free approaches are neither designed nor well-adapted to address functionally defined hypotheses in presence of highly multivariate phenotype data. Also new methods of multivariate phenotype analysis [18], [19] aim at extended screening rather than at addressing functionality. Functionality, in turn, is most straightforward to translate into a formally testable hypothesis by a regression model. Regression-based multivariate methods, however, which include classic multivariate linear regression analysis as well as alternative approaches like partial least squares (PLS) regression [20], are not frequently used to analyze multivariate phenotype data in biomedicine. The likely reason is that all regression-based methods are principally designed to test dependency, which in the multivariate case extends to a modeled best-fitting dependency structure between two sets of variables. With large empiric phenotypic datasets resulting from high-throughput methods, however, which are not obtained following specific prior expectations or hypotheses but rather defined by their mere practical accessibility, such multivariate dependency modeling is in most cases obviously inadequate. More adequate and promising in many instances would be an exploratory approach that does not depend on modeling a dependency structure of phenotype data but that is still capable of addressing functionally defined hypotheses. Such an approach is coreferentiality: to test whether hypothesis-defined variables share their *relatedness* to empiric multivariate phenotype data.

It may be argued here that an analogous approach of testing the relatedness among multiple independent X variables could theoretically also be undertaken in the frame of a regression model, and that its performance should be assessed both with and without modeling a phenotype dependency structure. However, this is not easily possible since multivariate regression analysis as it exists provides no adequate and testable criterion for the relatedness of two X variables in respect to a set of phenotypic Y variables. The only criterion that addresses relations between X variables in a linear regression model is collinearity, also called multicollinearity [21]. Collinearity, however, is not defined as relatedness, but rather as the capacity of X variables to replace each other in the explanation of Y, and is practically used to detect and avoid technical problems such as overfitting and matrix singularity, but not for data analysis. Particularly, (multi)collinearity is not formulated as a criterion for statistical testing. Therefore, although it is superficially similar, it has a quite different character than coreferentiality, and is in its existing definition clearly inadequate to address the same question.

Another method that may be discussed as a possible alternative is canonical correlation analysis. Supplementing a multivariate linear regression model with an analysis for canonical variates, canonical correlation provides an interpretation in terms of multiple independently testable orthogonal levels of relatedness. Accordingly, a multivariate regression model with two X variables, analogous to the situation where coreferentiality is tested, can contain either one or two significant canonical correlations. If it contains only one that has an impact of both X_1_ and X_2_, this points indeed to their coreferentiality. However, also canonical correlation analysis does not provide a formal criterion to test this. The significance of the canonical variates alone is not adequate: if both X are coreferential, their effects on the phenotypes Y will be represented in one canonical correlation, but this does not explicitly prevent a second one from being significant, which could represent a separate non-parallel side effect of one of the two X variables.

PLS regression seeks to identify “latent” variables in analogy to canonical variates by an alternative method [20], but is principally dedicated to the same goal of explaining a set of dependent Y variables as all regression-based methods. In summary, canonical correlation or similar methods could possibly be extended to test for an analog of coreferentiality. To the knowledge of the author, however, there is no explicit test for the parallellity between X variables that could be directly compared.

It can finally be stated that the non-regression-based coreferentiality approach proposed here appears better adapted than regression-based methods to address functional hypotheses in respect to empiric multivariate phenotype data in an exploratory manner. It furthermore has the merit of methodic parsimony in avoiding to unnecessarily model a dependency structure, but rather addressing indirect relatedness without any model assumption. Like classic bivariate direct correlation, this naturally includes the possibility of bidirectional effects. Accordingly, the nature of the tested effect itself is an undirected two-variable relation, which is likely more adequate to represent at least some useful hypotheses than are unidirectional dependencies as they are modeled in regression-based approaches. Particular hypotheses of this type in the context of a complex immune phenotype, particularly the role of a naturally bidirectional cytokine-receptor interaction, have largely motivated the development of the coreferentiality method. The corresponding research study is explicitly described in our accompanying paper [2], which may further illustrate the practical use and possible perspectives of the coreferentiality approach. Using this criterion, we could not only confirm a functional relatedness of effect pairs (e.g., specific autoantibodies and T-cell regulation), but also plausibly model the effect of a specific cytokine-receptor interaction in relation to broad-scale antibody profiles that served as reference data. The same approach can easily be applied to many other questions, including other types of multiparametric data. Among them, particularly data derived from mRNA expression arrays contain a potential information depth that should be at least comparable to that of the antibody profiles that we have used, and appear most promising to serve as reference data to study similar functionally defined bivariate hypotheses.

Another perspective of the described approach may be the functional interpretation of genetic variation particularly in human studies. In our accompanying publication [2], we were able to model also genetic effects and to bring them into a plausible functional context. This suggests that the wealth of available information on genetic variation particularly in human populations can indeed be directly used to interpret and model functionality. More systematically applied, this may open a new perspective of physiologic modeling, particularly in contexts that are not accessible to focused experimentation. It could also pave a new way to systematic subphenotype analysis, which has become more difficult in human genetic studies since traditional linkage analysis was replaced by genome-wide association. Other than genetic subphenotype analysis, however, the coreferentiality approach does not study the genetics of predefined subphenotypes, but constructs them following functional hypotheses using genetic information, which may be a promising alternative to investigate complex phenotypes.

## Materials and Methods

Data simulation and analysis was performed on a Macintosh computer with the software IgorPro (WaveMetrics), with particular procedures programmed for this purpose.

## References

[1] Rencher AC (1995). Methods of Multivariate Analysis.

[2] Fesel C, Barreto M, Ferreira RC, Costa N, Venda LL (2011). Compensatory T-Cell Regulation in Unaffected Relatives of SLE Patients, and Opposite IL-2/CD25-Mediated Effects Suggested by Coreferentiality Modeling.. PLoS One.

[3] Kempthorne O (1955). The randomization theory of experimental inference.. J Am Statist Assoc.

[4] Ludbrook J (1994). Advantages of permutation (randomization) tests in clinical and experimental pharmacology and physiology.. Clin Exp Pharmacol Physiol.

[5] Rasmussen JL (1989). Computer-intensive correlational analysis - bootstrap and approximate randomization techniques.. Brit J Math Statist Psychol.

[6] Allison DB, Cui XQ, Page GP, Sabripour M (2006). Microarray data analysis: from disarray to consolidation and consensus.. Nat Rev Genet.

[7] Nobrega A, Haury M, Grandien A, Malanchere E, Sundblad A (1993). Global Analysis of Antibody Repertoires. 2. Evidence For Specificity, Self-Selection and the Immunological Homunculus of Antibodies in Normal Serum.. Eur J Immunol.

[8] Haury M, Grandien A, Sundblad A, Coutinho A, Nobrega A (1994). Global Analysis of Antibody Repertoires .1. an Immunoblot Method For the Quantitative Screening of a Large Number of Reactivities.. Scand J Immunol.

[9] Mouthon L, Nobrega A, Nicolas N, Kaveri SV, Barreau C (1995). Invariance and Restriction Toward a Limited Set of Self- Antigens Characterize Neonatal Igm Antibody Repertoires and Prevail in Autoreactive Repertoires of Healthy-Adults.. Proc Natl Acad Sci USA.

[10] Sundblad A, Ferreira C, Nobrega A, Haury M, Ferreira E (1997). Characteristic generated alterations of autoantibody patterns in idiopathic thrombocytopenic purpura.. J Autoimmun.

[11] Fesel C, Coutinho A (1998). Dynamics of serum IgM autoreactive repertoires following immunization: strain specificity, inheritance and association with autoimmune disease susceptibility.. Eur J Immunol.

[12] Sharshar T, Lacroix-Desmazes S, Mouthon L, Kaveri S, Gajdos P (1998). Selective impairment of serum antibody repertoires toward muscle and thymus antigens in patients with seronegative and seropositive myasthenia gravis.. Eur J Immunol.

[13] Fesel C, Coutinho A (1999). Structured reactions of serum IgM repertoires to immunization are dependent on major histocompatibility complex genes.. Scand J Immunol.

[14] Fesel C, Coutinho A (2000). Serum IgM repertoire reactions to MBP/CFA immunization reflect the individual status of EAE susceptibility.. J Autoimmun.

[15] Santos-Lima EC, Vasconcellos R, Reina-San-Martin B, Fesel C, Cordeiro-da-Silva A (2001). Significant association between the skewed natural antibody repertoire of Xid mice and resistance to Trypanosoma cruzi infection.. Eur J Immunol.

[16] Silva S, Correia C, Fesel C, Barreto M, Coutinho AM (2004). Autoantibody repertoires to brain tissue in autism nuclear families.. J Neuroimmunol.

[17] Fesel C, Goulart LF, Neto AS, Coelho A, Fontes CJF (2005). Increased polyclonal immunoglobulin reactivity toward human and bacterial proteins is associated with clinical protection in human Plasmodium infection.. Malaria J 4:Article.

[18] Yang QO, Wu HS, Guo CY, Fox CS (2010). Analyze Multivariate Phenotypes in Genetic Association Studies by Combining Univariate Association Tests.. Genet Epidemiol.

[19] Medland S, Neale MC (2010). An integrated phenomic approach to multivariate allelic association.. Eur J Hum Genet.

[20] Abdi H (2010). Partial least squares regression and projection on latent structure regression (PLS-Regression).. Wiley Interdisciplinary Reviews: Computational Statistics.

[21] O'Brien RM (2007). A caution regarding rules of thumb for variance inflation factors.. Quality & Quantity.

